# Supplementary material for: Structural relationship between the putative hair cell mechanotransduction channel TMC1 and TMEM16 proteins
Source: eLife. 2018 Jul 31;7:e38433. doi: 10.7554/eLife.38433 (PMC6067890; doi:10.7554/eLife.38433)
Supplement: Transparent reporting form [file elife-38433-transrepform.pdf]

1st Floor
24 Hills Road
Cambridge CB2 1JP, UK

P 01223 855340
W elifesciences.org
T @elife

eLife's transparent reporting form

We encourage authors to provide detailed information *within their submission* to facilitate the interpretation and replication of experiments. Authors can upload supporting documentation to indicate the use of appropriate reporting guidelines for health-related research (see EQUATOR Network), life science research (see the BioSharing Information Resource), or the ARRIVE guidelines for reporting work involving animal research. Where applicable, authors should refer to any relevant reporting standards documents in this form.

If you have any questions, please consult our Journal Policies and/or contact us: editorial@elifesciences.org.

Sample-size estimation

- You should state whether an appropriate sample size was computed when the study was being designed
- You should state the statistical method of sample size computation and any required assumptions
- If no explicit power analysis was used, you should describe how you decided what sample (replicate) size (number) to use

Please outline where this information can be found within the submission (e.g., sections or figure legends), or explain why this information doesn't apply to your submission:

This information can be found in the Material and Methods section and figure legends 5, 7, 8 and 9. A sample size of 50-100 hair cells was used for quantification of the fluorescence intensity at the hair cell body, which is larger than commonly used in the field.

Replicates

- You should report how often each experiment was performed
- You should include a definition of biological versus technical replication
- The data obtained should be provided and sufficient information should be provided to indicate the number of independent biological and/or technical replicates
- If you encountered any outliers, you should describe how these were handled
- Criteria for exclusion/inclusion of data should be clearly stated
- High-throughput sequence data should be uploaded before submission, with a private link for reviewers provided (these are available from both GEO and ArrayExpress)

Please outline where this information can be found within the submission (e.g., sections or figure legends), or explain why this information doesn't apply to your submission:

This information can be found in the Material and Methods section.
At least three technical replication were performed for each experiment. For each technical replication, a minimum of three biological replicates (cochleae) were used in each experimental condition to control experimental variability and assure an appropriate sample size.
For quantification of the hair cell fluorescence intensity, every hair cell displayed in each image, including the ones that did not show dextran uptake, was included.
Non normalized, background subtracted mean fluorescence intensity and standard deviation are shown for each cell.

eLife Sciences Publications, Ltd is a limited liability non-profit non-stock corporation incorporated in the State of Delaware, USA, with company number 5030732, and is registered in the UK with company number FC030576 and branch number BR015634 at the address 1st Floor, 24 Hills Road, Cambridge CB2 1JP | August 2014

Statistical reporting

- Statistical analysis methods should be described and justified
- Raw data should be presented in figures whenever informative to do so (typically when N per group is less than 10)
- For each experiment, you should identify the statistical tests used, exact values of N, definitions of center, methods of multiple test correction, and dispersion and precision measures (e.g., mean, median, SD, SEM, confidence intervals; and, for the major substantive results, a measure of effect size (e.g., Pearson's r, Cohen's d)
- Report exact p-values wherever possible alongside the summary statistics and 95% confidence intervals. These should be reported for all key questions and not only when the p-value is less than 0.05.

Please outline where this information can be found within the submission (e.g., sections or figure legends), or explain why this information doesn't apply to your submission:

All statistical analysis were performed with the GraphPad Prism 7 software and are indicated in the corresponding figure legends of Figure 5, 7, 8, and 9.
One way ANOVA with Turkey's multiple comparison was used to analyze the data shown in Figure 5, 8 and 9, and unpaired t-test for the data shown in Figure 7. These analysis are also included in the additional Figure5-, Figure7-, Figure8- and Figure9-SourceData files.

(For large datasets, or papers with a very large number of statistical tests, you may upload a single table file with tests, Ns, etc., with reference to sections in the manuscript.)

Group allocation

- Indicate how samples were allocated into experimental groups (in the case of clinical studies, please specify allocation to treatment method); if randomization was used, please also state if restricted randomization was applied
- Indicate if masking was used during group allocation, data collection and/or data analysis

Please outline where this information can be found within the submission (e.g., sections or figure legends), or explain why this information doesn't apply to your submission:

This information can be found in the Material and Methods section.
Six day old litter-mate mice were used for each experimental condition.
Data collection and analysis of experiments performed with TMC1/TMC2 dKO mice were conducted blind.

Additional data files ("source data")

- We encourage you to upload relevant additional data files, such as numerical data that are represented as a graph in a figure, or as a summary table
- Where provided, these should be in the most useful format, and they can be uploaded as "Source data" files linked to a main figure or table
- Include model definition files including the full list of parameters used
- Include code used for data analysis (e.g., R, MatLab)
- Avoid stating that data files are "available upon request"

Please indicate the figures or tables for which source data files have been provided:

Mean fluorescence intensity data represented in Figure 5D, 7B, 8B and 9B graphs have been uploaded as the corresponding FigureX-SourceData file.
